# Supplementary material for: Differences and similarities between human and chimpanzee neural progenitors during cerebral cortex development
Source: eLife. 2016 Sep 26;5:e18683. doi: 10.7554/eLife.18683 (PMC5110243; doi:10.7554/eLife.18683)
Supplement: Figure 5—source data 1. — Numerical values in minutes for the duration of all mitotic phases ± SEM used in the graphs in Figures 5, 6 and 7, in Figure 5—figure supplement 1, 2 and 3, and in Figure 6—figure supplement 1. DOI: http://dx.doi.org/10.7554/eLife.18683.016 [file elife-18683-fig5-data1.docx]

**Figure 5-Source data 1**

| \| **Cell type** \| **Prophase** \| **Prophase + metaphase** \| *Congression*  *(“Prometaphase”)* \| *Metaphase plate*  *(“Metaphase”)* \| **Anaphase** \| **Telophase** \| \| --- \| --- \| --- \| --- \| --- \| --- \| --- \| \| **Hum. dev.**  **neoc.** \| 21.09 ± 0.40 \| 30.63 ± 0.88 \| 17.46 ± 0.58 \| 13.49 ± 0.44 \| 10.08 ± 0.23 \| 19.38 ± 0.42 \| \| **Hum. org. D30** \| 19.95 ± 0.46 \| 28.64 ± 0.75 \| 16.62 ± 0.60 \| 12.02 ± 0.36 \| 9.32 ± 0.2 \| 19.83 ± 0.29 \| \| **Chimp. Org. D30** \| 20.48 ± 0.43 \| 24.37 ± 0.48 \| 16.03 ± 0.6 \| 8.40 ± 0.36 \| 9.29 ± 0.19 \| 19.25 ± 0.3 \| \| **Orang. Org. D30** \| 21.39 ± 0.88 \| 22.75 ± 1.39 \| 14.88 ± 1.26 \| 7.88 ± 0.75 \| 10.15 ± 0.29 \| 19.06 ± 0.8 \| \| **Hum. org. D52** \| 20.21 ± 0.43 \| 25.72 ± 0.98 \| 15.63 ± 0.80 \| 10.09 ± 0.39 \| 9.45 ± 0.29 \| 19.49 ± 0.38 \| \| **Chimp. Org. D52** \| 20.2 ± 0.58 \| 23.49 ± 0.84 \| 14.59 ± 0.77 \| 8.90 ± 0.38 \| 9.90 ± 0.23 \| 20.1 ± 0.43 \| \| **Mouse dev. neoc.** \| 19.8 ± 0.4 \| 14.35 ± 0.36 \| 9.09 ± 0.38 \| 5.33 ± 0.13 \| 6.63 ± 0.11 \| 19.26 ± 0.35 \| \| *Mouse*  *Tis21::GFP–* \| 20.01 ± 0.62 \| 15.44 ± 0.69 \| 9.8 ± 0.56 \| 5.64 ±0.23 \| 6.62 ± 0.15 \| 18.9 ± 0.51 \| \| *Mouse*  *Tis21::GFP +* \| 19.56 ± 0.5 \| 13.52 ± 0.52 \| 8.54 ± 0.42 \| 4.98 ± 0.24 \| 6.64 ± 0.16 \| 19.69 ± 0.46 \| \| **Hum. iPSCs** \| 19.68 ± 0.46 \| 22.15 ± 0.51 \| 12.49 ± 0.42 \| 9.67 ± 0.38 \| 8.62 ± 0.18 \| 19.4 ± 0.59 \| \| **Chimp. iPSCs** \| 20.42 ± 0.5 \| 21.28 ± 0.56 \| 11.92 ± 0.4 \| 9.36 ± 0.4 \| 8.6 ± 0.12 \| 19.74 ± 0.37 \| \| **Hum. B cells** \| 18.5 ± 0.67 \| 20.81 ± 0.77 \| 11.76 ± 0.63 \| 9.05 ± 0.1 \| 7.93 ± 0.2 \| 19.13 ± 0.51 \| \| **Chimp. B cells** \| 18.17 ± 0.65 \| 21.36 ± 0.82 \| 12.9 ± 0.76 \| 8.47 9.05 ± 0.43 \| 8.57 ± 0.23 \| 18.57 ± 0.73 \| |
| --- | --- | --- | --- | --- | --- | --- | --- | --- | --- | --- | --- | --- | --- | --- | --- | --- | --- | --- | --- | --- | --- | --- | --- | --- | --- | --- | --- | --- | --- | --- | --- | --- | --- | --- | --- | --- | --- | --- | --- | --- | --- | --- | --- | --- | --- | --- | --- | --- | --- | --- | --- | --- | --- | --- | --- | --- | --- | --- | --- | --- | --- | --- | --- | --- | --- | --- | --- | --- | --- | --- | --- | --- | --- | --- | --- | --- | --- | --- | --- | --- | --- | --- | --- | --- | --- | --- | --- | --- | --- | --- | --- | --- | --- | --- | --- | --- | --- | --- |
